# Five Years' Experience of Surgery in Exophthalmic Goitre
*Paper read at a meeting of the Bath and Bristol Branch of the British Medical Association, 28th May, 1930.


**Published:** 1930

**Authors:** A. Rendle Short

**Affiliations:** Surgeon, Bristol Royal Infirmary


### FIVE YEARS' EXPERIENCE OF SURGERY IN EXOPHTHALMIC GOITRE.*

BY

A. Rendle Short, B.Sc., M.D., B.S., F.R.C.S.,

*Surgeon, Bristol Royal Infirmary.*

---

Until a few years ago the treatment of exophthalmic goitre was in a very unsatisfactory position. The cases seemed to differ from one another in a puzzling manner. Various enthusiasts held a brief for some line or other of medical or X-ray treatment, and could point to a few remarkable successes which sustained their zeal, but a dispassionate inquiry revealed many failures. The two main indubitable factors in bringing about recovery were rest and time, but often the time was long and the result uncertain. Surgery also achieved some spectacular successes, but there were many half-cures and numerous dreadful disasters. Patients died on the table, or succumbed to hemorrhage or suffocation four or five hours afterwards, or passed into a condition of fatal hyperthyroidism with very high temperature, uncountable pulse, pitiable excitement, and a tremor that shook the bed. Of 23 cases treated surgically before the war at Guy's and St. Thomas's Hospitals, one-third died, another third were no better, and only one or two were really symptom-free. Even when, encouraged by the successful series published by Kocher and the Mayo Clinic, better surgery gave

* Paper read at a meeting of the Bath and Bristol Branch of the British Medical Association, 28th May, 1930.

better results, we were warned not to touch patients with glycosuria or auricular fibrillation.

The general results obtained by medical treatment may be judged by the excellent analyses published by Hale White and by Williamson and Mackenzie :—

RESULTS OF MEDICAL TREATMENT OF EXOPHTHALMIC GOITRE.

| | Total cases. | Died in hospital. Per cent. | Total. | Cured. Per cent. | Much better. Per cent. | Better. Per cent. | No better. Per cent. | Died since. Per cent. |
|---|---|---|---|---|---|---|---|---|
| 1. Hale White's cases (hospital) .. .. | 161 | 11 | 48 | 54 | — | 25 | 5 | 16 |
| 2. Hale White's cases (private) .. .. | 55 | 0 | 54 | 60 | — | 17 | 6 | 13 |
| 3. Williamson and Mackenzie .. | 56 | 0 | 56 | 18 | 20 | 30 | 8 | 24 |

It will be observed that roughly about half the cases got well, a quarter were better, 6 per cent. not improved, and the mortality was double that of women of the same age in the general community. At the best the progress is tedious, six to eighteen months being a very ordinary time for restoration to fair health, but many cases drag on for years. I have seen them after eleven and even eighteen years. The course is apt to be progressively downwards for a few months, then gradual improvement takes place, which may or may not go on to recovery. The best indication of progress, good or ill, is the body weight. If it is rising there is general improvement, and *vice versa.*

Whether medical treatment at the present day has improved on these results it is for the physicians to say, but I take leave to doubt it. Probably I do not see the cases that get well quickly; perhaps some do. I see plenty that have dragged on a semi-invalid existence for years, and I know of several that have died of the disease before operation was decided upon.

An important step forward was taken when Plummer, in America, drew a distinction between the different types of thyroid intoxication. It is true that all intermediate grades between two extremes may be met with, and there is sometimes room for difference of opinion as to what a particular case should be labelled, but the differentiation into types is nevertheless of real value. The first type is classical exophthalmic goitre, called *primary thyroid intoxication* by Dunhill. The patient is usually a woman between 20 and 45 (though I have at the present time a girl of 9 and a man of 60 under treatment). The thyroid is enlarged more or less uniformly, and does not contain rounded nodules; exophthalmos is present. These signs, together with the well-known symptoms of nervousness, excitability, tachycardia, fine tremor of the hands, and loss of flesh, all come on together. The goitre may be soft, but in old cases it is hard; it may be very vascular and pulsating; sometimes the thyroid is scarcely at all enlarged.

At the other extreme we see patients usually past 40 with a history of enlarged thyroid for many years, and on palpation we find one or more rounded adenomatous nodules in the gland. At operation some of these are found to be breaking down, partially fluid, or containing a colloid material. The symptoms of thyroid intoxication are just like those of classical exophthalmic goitre, but they have developed comparatively recently, long after the patient knew she had a goitre. Exophthalmos, however, is absent. The nodule in the thyroid may be so small that it can only be found by careful search, if at all. These cases are usually called "toxic adenoma." Dunhill classifies them as *secondary thyroid intoxication.* He believes that the source of the thyroid intoxication, whether primary or secondary, is to be found in some stimulus brought to the gland, which may be psychical from some emotional upset, or may be septic, the original focus being found in the teeth, tonsils or elsewhere, or else it may be a febrile illness such as influenza. If the stimulus falls on a thyroid gland previously normal the result is primary thyroid intoxication or classical exophthalmic goitre. If the thyroid already contains adenomata, a secondary thyroid intoxication results. It is quite true that psychical, febrile or septic stimuli may make a case of Graves' disease worse, or cause a latent case to become obvious, and removal of the same will often benefit the patient, up to a point, but this theory scarcely seems to do justice to the fact that removal of a single degenerated adenoma will often cure the symptoms. Also, patients with thyroid intoxication are no more subject to psychical, septic or febrile upsets than the general community.

In the present address I have rather loosely grouped all cases of thyroid intoxication together under the title of exophthalmic goitre, as being more familiar to English ears. Cases of non-toxic parenchymatous, adenomatous or malignant goitre are not included.

This classification has a real clinical value, not annulled by the fact that intermediate grades are frequently seen. Classical exophthalmic goitre, primary thyroid intoxication, has a definite tendency in about half the cases to get well. As a rule, and for a brief period, it is favourably influenced by rest in bed, and the administration of Lugol's iodine, ten drops twice a day. It is true that the benefit soon wears off, and it is probably best to keep it in reserve for the week or two previous to an operation, when the effect may be very striking and valuable.

Toxic adenoma has no tendency to get well, and is but little improved by iodine or other remedies ; it is likely to go on to permanent damage to the cardiac muscle with auricular fibrillation. Glycosuria may be seen with either type of thyroid intoxication ; so may mental changes. Toxic adenoma ought always to be operated on, the presence of auricular fibrillation or glycosuria making the indications for removal not less but more urgent, and the risks of operation are very small, if the heart is not yet gravely diseased.

The proper place for surgery in classical exophthalmic goitre is not so easy to allocate. Five years ago we made it a rule to allow twelve months from the onset of symptoms before advising operation. During that year medical or X-ray treatment should be given an opportunity to effect a cure. Lately, encouraged by experience of the safety of operation and the practical certainty of benefit, we have been offering, if not advising, surgical treatment to quite early cases. Take, for example, a young woman earning her own living, seen recently, with symptoms of Graves' disease for three months, getting worse. Which shall we offer her, a year's rest, with only a 50 per cent. chance at the end of that time of being well enough to do her work, or an operation, that holds out good prospect of making her fit in, say, two months ? It is not surprising that she, her relatives, and her doctor chose the latter, and she is doing very well.

We turn, then, to the question of the risks and benefits of surgery to-day for thyroid intoxication. Dunhill, with his immense experience, reports a mortality of only 2·7 per cent. Even of 100 cases of auricular fibrillation only 9 per cent. died. Regular rhythm returned in 48 patients spontaneously, and in 32 more with the aid of quinidine.

During the past five years I have operated 62 times on 52 patients for classical exophthalmic goitre or for toxic adenoma. There have been, of course, a number of operations for ordinary adenomatous or parenchymatous goitre besides. The patients had been ill for over a year in all cases, except one just mentioned, and often for many years. Four patients died in hospital. One of these was very ill with auricular fibrillation, and operation was rather a forlorn hope. She died next day, and the post-mortem showed broncho-pneumonia and a fatty liver. Another survived ten days, but finally succumbed, and was found to have abscesses of the kidney, the culture from which showed *B. coli*. Both these cases may be regarded as marked for death anyhow. The others died of reactionary hemorrhage, a risk which can be practically eliminated by the measures we are now taking against it. More than half the cases were of classical exophthalmic goitre.

The procedure adopted has been as follows. The patient is kept in bed in the nursing home or hospital for at least a week. During this time Dr. Herapath has usually seen them, and often an electrocardiogram has been taken. In cases of the classical Graves' disease type Lugol's iodine is given. The anæsthetic has been given in practically all my cases by Dr. L. A. Moore. It has usually been open ether, but in our last eight or ten cases we have used avertin and a local anæsthetic, with or without a drachm or two of ether. To obtain a real cure it is necessary in cases of toxic adenoma to remove the degenerating nodules, and in exophthalmic goitre to take away five-sixths of the total gland substance. Sometimes this can be done safely at one operation. More frequently it is better to content oneself with a hemi-thyroidectomy, and to remove part of the remaining lobe about three months later, when the patient is likely to be greatly improved. In one case it was thought best at the first intervention merely to tie the superior thyroid arteries. The steps of the operation are as follows: A collar incision is made, with reflection of muscles and fascia to expose the goitre. The superior thyroid artery is ligatured and re-ligatured. The corresponding lobe of the thyroid gland is enucleated. Then the branches of the inferior thyroid artery are ligatured and re-ligatured close to the gland, and the goitre is resected on that side, leaving a portion at the back and adjacent to the trachea. The raw surface is infolded and sutured, all small vessels are transfixed and tied, and the patient is allowed to come round to cough and strain, so as to demonstrate hæmostasis; the wound is then sewn up with a stab drain. A special nurse is in charge for twelve hours. After that time has elapsed the risks are almost past.

Ill consequences are unusual. I have not seen tetany follow. A few cases have had weakness of the voice from recurrent laryngeal nerve palsy; this has more than once come on late, and seems to be due to involvement in scar tissue, not section. Three patients have had fatal illnesses months after apparent recovery, and probably due to some intercurrent disease. One patient suffering from chronic insomnia was greatly benefited physically, but the result was largely spoiled by the persistent inability to sleep. Another, an old man, did well for years, then became insane. As far as I know, all the rest are either quite symptom-free, or well enough to lead a more or less normal life.

Some are not perfect, because having had a hemithyroidectomy, they are so well that they did not desire a second operation. Cure is merely a question of removing enough thyroid. The pulse-rate usually returns to normal about ten days after the operation.

As Walton has pointed out, these patients suffer badly from heat, and it is not wise to operate during the few weeks of really hot weather that we usually get in July.

Patients with a thyroid adenoma ought not to be left until degeneration giving rise to toxic symptoms has set in. By removal at an earlier period this risk, and that of malignancy, can and ought to be avoided.

To sum up, in our opinion all cases of toxic adenoma of the thyroid should be operated on, and all cases of classical exophthalmic goitre after a year's duration, unless mentally deranged. There is a good deal of reason for operating also in early cases. Auricular fibrillation and glycosuria will probably clear up after subtotal thyroidectomy.

